# Can the genetic polymorphisms of the folate metabolism have an influence in the polycystic ovary syndrome?

**DOI:** 10.20945/2359-3997000000167

**Published:** 2019-08-28

**Authors:** Tayssia Beatriz dos Santos, Helena Korres de Paula, Marly Aparecida Spadotto Balarin, Roseane Lopes Silva-Grecco, Marco Fábio Prata Lima, Elisabete Aparecida Mantovani Rodrigues de Resende, Mariana Kefalas Oliveira Gomes, Mariangela Torreglosa Ruiz Cintra

**Affiliations:** 1 Universidade Federal do Triângulo Mineiro Universidade Federal do Triângulo Mineiro Uberaba MG Brasil Universidade Federal do Triângulo Mineiro (UFTM), Uberaba, MG, Brasil

**Keywords:** Polycystic ovary syndrome, folic acid, polymorphism

## Abstract

**Objective:**

To investigate the association of the genetic variants of the folate metabolism genes (*MTHFR* C677T; *MTHFR* A1298C; *MTR* A2756G; *MTRR* A66G and *RFC-1* A80G) with the development of polycystic ovary syndrome (PCOS).

**Subjects and methods:**

This study included 203 women (99 women with PCOS and 104 controls). The genotyping was performed by PCR-RFLP. Chi-squared test and multiple logistic regression were used in the statistical analysis. Haplotype analysis was conducted using the SNPstat program. The results were presented in *odds ratio* (OR) and confidence interval of 95% (CI-95%), with a significance level of 5% (*p* ≤ 0.05).

**Results:**

The genotypic distribution of the *RFC-1* A80G polymorphism showed significant difference between the two groups, showing that the heterozygous genotype (AG genotype) was most frequent in controls. The polymorphic homozygous (GG genotype) of *MTRR* A66G polymorphism were most frequent in controls. The T-C haplotype *MTHFR* C677T and A1298C polymorphisms were more frequent in the control group (OR = 0.19; CI 95% — 0.04 to 0.93 e p = 0.042). The multivariate analysis evidenced that family history of PCOS was more frequent in the PCOS group (OR = 3.29; CI 95% — 1.48 to 7.31; p = 0.003).

**Conclusion:**

In our casuistry, the polymorphic homozygous of *MTRR* A66G polymorphism gene and heterozygous of *RFC*-1 A80G polymorphism gene, the haplotype T-C C677T and A1298C polymorphisms of *MTHFR* gene, can be associated with protective factors for the disease.

## INTRODUCTION

The Polycystic Ovary Syndrome (PCOS) is characterized by the presence of polycystic ovaries morphology, oligo or chronic anovulation and hyperandrogenism (1,2).

The etiology of PCOS is unclear, but this syndrome is a multifactorial disease that results from complex interactions between genetic and environmental factors (3). One study performed in 1968 showed the possible effect of folic acid on ovarian function in rats, evidencing that a deficiency or an excess of folic acid partially inhibited ovulation (4). The effect of folate deficiency was also studied on the cytomorphology and the kinetics of proliferation of ovarian granulosa cells, and the epithelial cells lining the uterus, cervix and vagina of six sexually mature female rhesus monkey and showed degeneration of Graffian follicles with an increase in atretic and cystic follicles, accompanied by a depletion of granulosa cells and reduction or even absence of corpora lutea (5). The homocysteine is produced in all cells and can be produced by folate metabolism that is reduced to tetrahydrofolate which is then converted to 5, 10-methylenetetrahydrofolate (6). Several studies confirmed the presence of increased serum Hcy concentration in PCOS patients (7).

The enzymes methylenetetrahydrofolate reductase (MTHFR), methionine synthase (MTR), methionine synthase reductase (MTRR) and reduced folate carrier 1 (RFC-1) participate in the folate metabolism. Single nucleotide polymorphisms (SNPs) can lead to modifications in enzymatic activities involved in the metabolic pathway of folate and may be implicated in the etiology of the PCOS, mainly in epigenetic changes such as alterations in DNA methylation pattern (4-6,8). Several literature reports showed that the *MTHFR* C667T and A1298C, *MTR* A2756G, *MTRR* A66G and *RFC-1* A80G SNPs are genetic factors that can modify the activities of the folate metabolism enzymes (9-12).

Several studies evaluated the association between PCOS and *MTHFR* polymorphisms and found contradictory results. However, the study of Fu and colleagues showed that the T allele of MTHFR C677T polymorphism is associated with the PCOS development (13).

Considering the evidences showed in several studies indicating that the genetic polymorphisms can have influence in PCOS and the lack of studies in the Brazilian population, the aim of this study was to investigate the association of the genetic variants of the folate metabolism genes (*MTHFR C677T* – rs1801133, *MTHFR A1298C* – rs1801131, *MTR A2756G* – rs1805087, *MTRR* A66G – rs1801394 and *RFC-1* A80G – rs1051266 polymorphisms with the development of PCOS).

## SUBJECTS AND METHODS

### Casuistry

This case-control study was approved by the Ethics Committee of the Federal University of Triângulo Mineiro (UFTM) (Protocol 1796), with all patients signing the Informed Consent Term.

All the women in this study were availed by specialists of a research group from the Gynecology and Obstetrics and the Adrenal and Gonadal Endocrinology sectors of the Clinics Hospital of UFTM, Uberaba, Minas Gerais, Brazil. Participants inclusion in the study occurred in the period from 2012 to 2016.

This study included 203 women **(**99 women with PCOS and 104 controls). PCOS patients were diagnosed using the Rotterdam criteria. The control group consisted of women in reproductive age, with no current or superior use of contraceptives for 3 months and no history or signs of PCOS. The exclusion criteria consisted of: presence of Cushing’s syndrome, congenital adrenal hyperplasia, thyroid dysfunction, hyperprolactinemia, diabetes mellitus, adrenal or ovarian tumors, use of anti-androgens, statins, glucocorticoids or medications for infertility.

### Molecular analysis

The genomic DNA was extracted from peripheral blood leukocytes according to the method of Phenol: Chloroform: Isoamyl (14) and storage at 4^o^C. The genotyping of the *MTHFR C677T* (rs1801133), *MTHFR A1298C* (rs1801131), *MTR A2756G* (rs1805087), *MTRR* A66G (rs1801394) and *RFC-1* A80G (rs1051266) polymorphisms were analyzed by Polymerase Chain Reaction and Restriction Fragment Length Polymorphism (PCR-RFLP) using protocols previously described (15-18).

### Statistical analysis

Statistical analyses were performed using Stacts Direct and BioEstat version 3.0. Chi-squared test and multiple logistic regression were used in the statistical analysis.

Comparisons between the differences of genotypic distributions and Hardy-Weinberg equilibrium (HWE) were made by chi-square test. The multiple regression logistic test was used to determine the effects of variables in PCOS. The model included smoking habit (reference: no), alcohol habit (reference: no), family history of PCOS (reference: no), and the polymorphisms (reference: homozygous wild type).

The binary logistic regression model adjusted for age was used to assess the association between polymorphisms and development of PCOS using the SNPStats program (available at: http://bioinfo.iconcologia.net/SNPstats_web). The effects of the polymorphisms were evaluated in the following models: (1) codominant (homozygous wild type *vs* heterozygous *vs* polymorphic homozygote); (2) dominant (homozygous wild type *vs* heterozygous *plus* polymorphic homozygous); (3) recessive (polymorphic homozygous *vs* homozygous wild type *plus* heterozygous) and (4) overdominant (homozygous wild type *plus* polymorphic homozygous *vs* heterozygous). The *MTHFR* haplotype was inferred using the SNPStats program, which checked population frequency estimates of the haplotype.

The software G POWER 3.1 was used to verify the statistical power of the study and presented 95%, post hoc.

The results were shown as odds ratios (OR) and 95% confidence interval (CI 95%). The level of significance was set at 5% (p ≤ 0.05).

## RESULTS

In this study, sociodemographic data, including age, smoking and alcohol habits and historical family PCOS, were collected. Also, information as absence of pregnancy and menstrual irregularities, comorbidities, use of hormonal or non-hormonal medication, hyperandrogenic aspects, weight, height, BMI were collected and are shown in the Table 1. The clinical data were collected by follow-up in the medical records.

**Table 1:** Characterization of the study group with women with PCOS and controls

Variables	PCOS	Controls

	N/n (% or mean)	N/n (% or mean)
Age (years)	97 (26.06 ± 7.32*)	99 (31.73 ± 9.65*)
Absence of pregnancy	96/76 (79.17)	99/38 (38.38)
Menstrual irregularities	97/34 (35.05)	98/06 (06.12)
Family history of PCOS	95/37 (38.95)	100/20 (20.00)
Smoking habit	95/07 (07.37)	100/25 (25.00)
Alcohol habit	97/25 (25.77)	100/29 (29.00)
Comorbidities	94/44 (46.81)	98/29 (29.59)
Use of hormonal medication	95/54 (56.84)	100/09 (09.00)
Hirsutism	93/52 (55.91)	100/05 (05.00)
Acne	93/61 (65.59)	100/29 (29.00)
Skin Oiliness	93/73 (78.49)	100/49 (49.00)
Loss of hair	92/58 (63.04)	99/35 (35.35)
Weight (meters)	89 (1.63 ± 0.07*)	96 (1.62 ± 0.06*)
Height (kg)	91 (79.70 ± 22.45*)	96 (67.34 ± 14.65*)
BMI (kg/m^2^)	89 (29.72 ± 8.82*)	96 (25.44 ± 4.86*)
Overweight or obesity	/58 (65.17)	/48 (50.00)

* Mean of variable and standard deviation; N: total of women with the analyzed variable; n: number of women with the variable analyzed.

In the univariate analysis, the genotype distribution of *RFC-1* A80G polymorphism showed significant difference between the two groups (χ^2^ = 8.42; p = 0.01). For the other polymorphisms, the genotype distribution has not shown statistic difference between the cases and controls for *MTHFR* C677T (χ^2^ = 1.14; p = 0.56), *MTHFR* A1298C (χ^2^ = 0.04; p = 0.98), *MTR* A2756G (χ^2^ = 0.13; p = 0.57) and *MTRR* A66G polymorphisms (χ^2^ =3.47; p = 0.18).

With regard to the HWE, the genotypic frequencies of the *MTHFR* C677T (χ^2^ = 0.11; p = 0.74 cases; χ^2^ = 0.03; p = 0.86 controls) and *MTR* A2756G polymorphisms (χ^2^ = 0.0006; p = 0.98 cases; χ^2^= 0.33; p = 0.57 controls) were found in equilibrium. The *MTRR* A66G and *RFC-1* A80G polymorphisms showed equilibrium in controls (χ^2^ = 3.28; p = 0.07; χ^2^= 0.05; p = 0.83, respectively), but disequilibrium in cases (χ^2^ = 5.55; p = 0.02; χ^2^ = 14.01; p = 0.0002, respectively). The *MTHFR A1298C* polymorphism showed disequilibrium in cases and controls (χ^2^ = 8.33; p = 0.004; χ^2^ = 8.73; p = 0.003, respectively).

Table 2 shows the haplotypes that were constructed with the analysis of the *MTHFR* polymorphisms genes studied (C677T and A1298C). The C-A, T-A and C-C haplotype frequencies were similar between both groups. However, the T-C haplotype frequency showed significant difference between the PCOS group and control group (OR = 0.19; CI 95%: 0.04-0.93 and p = 0.042), with higher frequency in the control group.

**Table 2:** Haplotype frequencies of the *MTHFR* C677T and A1298C polymorphisms gene in women with PCOS and controls

Haplotype	PCOS Frequency	Control Frequency	O.R (CI 95%)	*p* value
C-A	0.38	0.39	1.00	-
T-A	0.29	0.29	1.07 (0.62 to 1.82)	0.82
C-C	0.31	0.26	1.53 (0.91 to 2.56)	0.11
T-C	0.02	0.07	0.19 (0.04 to 0.93)	0.042*

OR: odds ratio; CI: confidence interval. * p < 0.05.

In our study, the analysis of the association of the five genetic polymorphisms (the *MTHFR* C677T*, MTHFR* A1298C*, MTR* A2756G*, MTRR* A66G *and RFC-1* A80G) is presented in Table 3. The genotypes of 99 women with PCOS and 90 controls were adjusted for age according to the heritable models. The SNP *MTRR* A66G in the recessive model (OR = 0.34; CI 95%: 0.12-0.96; p = 0.035) showed a higher frequency of the recessive genotype in the control group. The SNP *RFC-1* A80G in the codominant model (OR = 0.42; CI 95%: 0.20-0.87; p = 0.049) and overdominant model (OR = 0.46; CI 95%: 0.25-0.88; p = 0.017) had higher frequency of the heterozygous genotype in the control group. These results may suggest an association of these polymorphisms as protective factors in PCOS development.

**Table 3:** Association of the *MTHFR* C677T, *MTHFR* A1298C, *MTR* A2756G, *MTRR* A66G and *RFC*-1 A80G polymorphisms gene with PCOS, adjusted for age

Model	Genotype		Case N (%)	Control N (%)	OR (CI 95%)	*p* value

*MTHFR* C677T						
Codominant	C/C	44 (47.30)		39 (43.30)	1.00	0.32
	C/T	42 (45.20)		39 (43.30)	0.90 (0.47-1.73)	
	T/T	07 (07.50)		12 (13.40)	0.45 (0.15-1.30)	
Dominant	C/C	44 (47.30)		39 (43.30)	1.00	0.45
	C/T-T/T	49 (52.70)		51 (56.70)	0.79 (0.43-1.46)	
Recessive	C/C-C/T	86 (92.50)		78 (86.70)	1.00	0.14
	TT	07 (07.50)		12 (13.40)	0.47 (0.17-1.30)	
Overdominant	C/C-T/T	51 (54.80)		51 (56.70)	1.00	0.87
	C/T	42 (45.20)		39 (43.30)	1.05 (0.57-1.95)	

***MTHFR* A1298C**						

Codominant	A/A	48 (51.60)		48 (53.30)	1.00	
	A/C	29 (31.20)		27 (30.00)	1.43 (0.70-2.94)	0.60
	C/C	16 (17.20)		15 (16.70)	1.26 (0.54-2.95)	
Dominant	A/A	48 (51.60)		48 (53.30)	1.00	
	A/C-C/C	45 (48.40)		42 (46.70)	1.36 (0.73-2.55)	0.33
Recessive	A/A-A/C	77 (82.80)		75 (83.30)	1.00	
	C/C	16 (17.20)		15 (16.70)	1.10 (0.49-2.48)	0.81
Overdominant	A/A-C/C	64 (68.80)		63 (70.00)	1.00	
	A/C	29 (31.20)		27 (30.00)	1.35 (0.68-2.67)	0.39

***MTR* A2756G**						

Codominant	A/A	59 (63.40)		54 (60.00)	1.00	0.71
	A/G	31 (33.30)		31 (34.40)	0.86 (0.45-1.64)	
	G/G	03 (03.20)		05 (05.60)	0.56 (0.12-2.60)	
Dominant	A/A	59 (63.40)		54 (60.00)	1.00	0.53
	A/G-G/G	34 (36.60)		36 (40.00)	0.82 (0.44-1.53)	
Recessive	A/A-A/G	90 (96.80)		85 (94.40)	1.00	0.49
	GG	03 (03.20)		05 (05.60)	0.59 (0.13-2.70)	
Overdominant	A/A-G/G	62 (66.70)		59 (65.60)	1.00	0.73
	A/G	31 (33.30)		31 (34.40)	0.89 (0.47-1.70)	

***MTRR* A66G**						

Codominant	A/A	33 (35.50)		25 (27.80)	1.00	0.09
	A/G	54 (58.10)		51 (56.70)	0.81 (0.41-1.60)	
	G/G	06 (06.50)		14 (15.60)	0.29 (0.09-0.92)	
Dominant	A/A	33 (35.50)		25 (27.80)	1.00	0.28
	A/G-G/G	60 (64.50)		65 (72.20)	0.69 (0.36-1.34)	
Recessive	A/A-A/G	87 (93.50)		76 (84.40)	1.00	0.035*
	GG	06 (06.50)		14 (15.60)	**0.34 (0.12-0.96)**	
Overdominant	A/A-G/G	39 (41.90)		39 (43.30)	1.00	0.77
	A/G	54 (58.10)		51 (56.70)	1.10 (0.59-2.04)	

***RFC-1* A80G**						

Codominant	A/A	36 (38.70)		25 (27.80)	1.00	0.049*
	A/G	28 (30.10)		45 (50.00)	**0.42 (0.20-0.87)**	
	G/G	29 (31.20)		20 (22.20)	0.79 (0.35-1.78)	
Dominant	A/A	36 (38.70)		25 (27.80)	1.00	0.066
	A/G-G/G	57 (61.30)		65 (72.20)	0.54 (0.28-1.05)	
Recessive	A/A-A/G	64 (68.80)		70 (77.80)	1.00	0.49
	GG	29 (31.20)		20 (22.20)	1.28 (0.64-2.56)	
Overdominant	A/A-G/G	65 (69.90)		45 (50.00)	1.00	0.017*
	A/G	28 (30.10)		45 (50.00)	**0.46 (0.25-0.88)**	

OR: odds ratio; CI: confidence interval. * p ≤ 0.05.

Table 4 shows the results of multiple logistic regression analysis in 85 women with PCOS and 84 controls to determine the effects of the variables. The smoking habit was more frequent in the control group (OR = 0.13; CI 95%: 0.04-0.41; p = 0.004). The PCOS family history was more frequent in the case group (OR = 3.29; CI 95%: 1.48-7.31; p = 0.003).

**Table 4:** Relation between risk factors for PCOS development and the genetic polymorphisms of the *MTHFR* C677T, *MTHFR* A1298C, *MTR* A2756G, *MTRR* A66G and *RFC*-1 A80G in women with PCOS and controls

Variable analyzed	PCOS N (%)	Control N (%)	O.R (CI 95%)	p value

Risk factors				
**Smoking Habit**				
Yes	05 (05.88)	23 (27.38)	0.13 (0.04-0.41)	0.004*
No	80 (94.11)	61 (72.62)		
**Alcohol Habit**				
Yes	20 (23.53)	24 (28.57)	0.96 (0.44-2.07)	0.91
No	65 (76.47)	60 (71.43)		
**Family History of PCOS**				
Yes	31 (36.47)	14 (16.67)	**3.29 (1.48-7.31)**	0.003*
No	54 (63.53)	70 (83.33)		

**GENETIC POLYMORPHISMS**				

***MTHFR* A1298C**			1.06 (0.53-2.13)	
AA	42 (49.41)	46 (54.76)		0.87
AC / CC	43 (50.59)	38 (45.24)		
***MTHFR* C677T**			1.01 (0.50-2.01)	
CC	40 (47.06)	37 (44.05)		
CT / TT	45 (52.94)	47 (55.95)		0.98
***MTR* A2756G**			0.85 (0.43-1.68)	
AA	52 (61.18)	51 (60.71)		0.64
AG / GG	33 (38.82)	33 (39.29)		
***MTRR* A66G**			0.67 (0.33-1.36)	
AA	32 (37.65)	23 (27.38)		0.26
AG / GG	53 (62.35)	61 (72.62)		
***RFC-1* A80G**			0.52 (0.25- 1.06)	
AA	35 (41.18)	24 (28.57)		0.07
AG / GG	50 (58.82)	60 (71.43)		

OR: odds ratio; CI: confidence interval. * p ≤ 0.05.

## DISCUSSION

This study evaluated the association of five genetic polymorphisms involved in the folate metabolism (*MTHFR* C677T, *MTHFR* A1298C, *MTR* A2756G*, MTRR* A66G and *RFC-1* A80G) in women with PCOS and controls in a Brazilian population. The analysis of polymorphisms of the folate metabolic pathway enzymes genes can provide relevant data on how these genetic variants may alter the structural and functional properties of the enzymes, and result in important alterations. The enzymes methylenetetrahydrofolate reductase enzyme (MTHFR), methionine synthase (MTR) and methionine synthase reductase (MTRR), are involved in folate metabolism, and polymorphisms identified in their genes can lead to the functional alterations and may alter the folate metabolism (7,19).

The present study tested HWE for the genotype frequencies of the five polymorphisms analyzed. The polymorphisms *MTHFR* A1298C were in disequilibrium in the cases and controls, and the polymorphisms *MTRR* A66G and *RCF-1* A80G presented disequilibrium in the case group. When the HWE was tested, the disequilibrium between cases and controls may be due to the random selection of the individuals studied, the adopted disease model and/or random changes in the genotype frequencies due to sampling errors (genetic drift). When there is no balance among the cases, it may be related to the disease (with a statistical difference in the genotypic frequency) or deletion of the deleterious allele by the action of natural selection (with no statistical difference in genotypic frequency). When the control individuals are in HW disequilibrium, it may mean that there is a protective effect (20-22).

In our results, there were no association between PCOS and the SNPs C677T and A1298C *MTHFR* gene. Table 5 summarizes the case-control studies with PCOS and *MTHFR* gene polymorphisms in different populations and shows that the results of the association of these polymorphisms are contradictory.

**Table 5:** Case-control studies with PCOS and MTHFR gene polymorphisms

Authors	Country	SNPs	Cases Controls		Conclusion
Wu and cols., 2016 (23)	China	*MTHFR* C677T *MTHFR* A1298C	244	257	No association
Szafarowska and cols., 2016 (24)	Poland	*MTHFR* C677T *MTHFR* A1298C	76	56	No association
Carlus and cols., 2016 (25)	India	*MTHFR* C677T	261	256	No association
Qi and cols., 2015 (26)	China	*MTHFR* C677T *MTHFR* A1298C	115	58	Association with *MTHFR* C677T and PCOS No association with *MTHFR* A1298C
Idali and cols., 2012 (27)	Iran	*MTHFR* C677T *MTHFR* A1298C	71	106	No association with *MTHFR* C677T and A1298C and PCOS Association with two SNPs *MTHFR* in women with PCOS plus recurrent pregnancy loss
Karadeniz and cols., 2010 (28)	Turkey	*MTHFR* C677T	86	70	No association
Choi and cols., 2009 (29)	Korea	*MTHFR* C677T	227	115	No association

This study evaluated the relationship between haplotypes of the *MTHFR* gene polymorphisms and PCOS. The haplotype T-C was more frequent in the controls. Other studies have not made this inference.

The present study has not found any association between the SNP *MTR* A2756G and PCOS. This result is in agreement with one study available in the literature that analyzed 46 women with PCOS and 25 controls, in two ethnicities: Caucasian (25 PCOS and 16 controls) and South Asian (21 PCOS and 9 controls), and the relation of MTR A2756G polymorphism with the biochemical, hematological and endocrinological parameters and plasma levels of homocysteine (8).

No association between SNP *MTRR* A66G and PCOS were observed in our study. However, the recessive inheritance model showed that GG genotype is more frequent in the control group and this result can suggest that the recessive homozygous can be related as a protective factor in our studied population. Another study showed association between the lower level of the total cholesterol and LDL cholesterol when compared to AA and AG genotypes (30).

In our study, the univariate analysis of the SNP *RFC-1* A80G showed significant difference between the cases and controls. The codominant and overdominant inheritance models showed that the AG genotype was more frequent in controls. Results of our study suggest that heterozygous individuals can show protector effect against PCOS. There are few studies in the literature about the *RFC-1*A80G polymorphism gene and some comorbidities, like hyperhomocysteinemia (31).

Regarding casuistry in this study, the multivariate analysis evidenced that family history of PCOS was more frequent in women with PCOS. This result shows that genetic factors can be related to the etiology of PCO0S, which is in accordance with other studies. A study carried out in 2006 evidenced that the syndrome has a hereditary basis, and that genetic factors are related with PCOS development (32). Other research, in 2010, investigated the genes susceptible to the development of PCOS in 502 families with daughters and sisters with PCOS and it concluded that some genes are related to the risk of developing the syndrome (33). Therefore, this information constitutes an important basis for justification of studies of susceptible genes in PCOS. This analysis also showed that smoking habits was more frequent in controls; this finding can be due to the treatment of PCOS in the case of female smokers includes quitting smoking. Discontinuation of cigarette smoking may result in decreased levels of androgens in the circulation. Likewise, smoking is a contraindication to the use of oral contraceptives, which are often prescribed to patients with PCOS (34,35).

In this study, we did not collect the level of homocysteine and data reference of the food supplementation from female participants. It was not possible to conduct the analysis of the biochemical factors and the SNPs studied because the laboratory data was performed using medical records. These facts constituted limitations to our study. However, this study is very relevant because there are few studies regarding polymorphisms in PCOS in the Brazilian population and this is the first study that analyzed the polymorphisms of folate metabolism in the syndrome.

In conclusion, in relation to casuistry, the polymorphic homozygous of MTRR A66G polymorphism gene and heterozygous of RFC-1 A80G polymorphism gene, the haplotype T-C C677T and A1298C polymorphisms of MTHFR gene, can be associated with protective factors for the disease.

## References

[1] Conway G, Dewailly D, Diamanti-Kandarakis E, Escobar-Morreale HF, Franks S, Gambineri A, et al. The polycystic ovary syndrome: a position statement from the European Society of Endocrinology. Eur J Endocrinol. 2014;171(4):P1-29. 10.1530/EJE-14-0253 24849517

[2] Rotterdam ESHRE/ASRM-Sponsored PCOS Consensus Workshop Group. Revised 2003 consensus on diagnostic criteria and long-term health risks related to polycystic ovary syndrome. Fertil Steril. 2004;81(1):19-25. 10.1016/j.fertnstert.2003.10.004 14711538

[3] Venkatesh T, Suresh PS, Tsutsumi R. New insights into the genetic basis of infertility. Appl Clin Genet. 2014;7:235-43. 10.2147/TACG.S40809 PMC4259396 25506236

[4] Willmott M, Bartosik DB, Romanoff EB. The effect of folic acid on superovulation in the immature rat. J Endocrinol. 1968;41(3):439-50. 10.1677/joe.0.0410439 5711115

[5] Mohanty D, Das KC. Effect of folate deficiency on the reproductive organs of female rhesus monkeys: a cytomorphological and cytokinetic study. J Nutr. 1982;112(8):1565-76. 10.1093/jn/112.8.1565 7097366

[6] Price BR, Wilcock DM, Weekman EM. Hyperhomocysteinemia as a Risk Factor for Vascular Contributions to Cognitive Impairment and Dementia. Front Aging Neurosci. 2018;10:350. 10.3389/fnagi.2018.00350 PMC6220027 30429785

[7] Grodnitskaya EE, Kurtser MA. Homocysteine metabolism in polycystic ovary syndrome. Gynecol Endocrinol. 2012;28(3):186-9. 10.3109/09513590.2011.589927 21793705

[8] Palep-Singh M, Picton HM, Yates ZR, Barth JH, Balen AH. Plasma homocysteine concentrations and the single nucleotide polymorphisms in the methionine synthase gene (MTR 2756A>G): Associations with the polycystic ovary syndrome an observational study. Eur J Obstet Gynecol Reprod Biol. 2008;138(2):180-6. 10.1016/j.ejogrb.2007.12.015 18281142

[9] Frosst P, Blom HJ, Milos R, Goyette P, Sheppard CA, Matthews RG, et al. A candidate genetic risk factor for vascular disease: a common mutation in methylenetetrahydrofolate reductase. Nat Genet. 1995;10(1):111-13. 10.1038/ng0595-111 7647779

[10] Weisberg I, Tran P, Christensen B, Sibani S, Rozen R. A second genetic polymorphism in methylenetetrahydrofolate reductase (MTHFR) associated with decreased enzyme activity. Mol Genet Metab. 1998;64(3):169-72. 10.1006/mgme.1998.2714 9719624

[11] Gaughan DJ, Kluijtmans LA, Barbaux S, McMaster D, Young IS, Yarnell JW, et al. The methionine synthase reductase (MTRR) A66G polymorphism is a novel genetic determinant of plasma homocysteine concentrations. Atherosclerosis. 2001;157(2):451-6. 10.1016/s0021-9150(00)00739-5 11472746

[12] Niedzielska E, Weçcławek-Tompol J, Matkowska-Kocjan A, Chybicka A. The influence of genetic RFC1, MS and MTHFR polymorphisms on the risk of acute lymphoblastic leukemia relapse in children and the adverse effects of methotrexate. Adv Clin Exp Med. 2013;22(4):579-84. 23986219

[13] Fu LY, Dai LM, Li XG, Zhang K, Bai Y. Association of methylenetetrahydrofolate reductase gene C677T polymorphism with polycystic ovary syndrome risk: a systematic review and meta-analysis update. Eur J Obstet Gynecol Reprod Biol. 2014;172:56-61. 10.1016/j.ejogrb.2013.10.001 24192663

[14] Sambrook J, Fritschi EF, Maniatis T. Molecular cloning: a laboratory manual. In: Sambrook J, Fritschi EF, Maniatis T. Cold spring harbor laboratory press. 2nd ed. New York: Cold Spring Harbor; 1989.

[15] van der Put NM, Gabreëls F, Stevens EM, Smeitink JA, Trijbels FJ, Eskes TK, et al. A second common mutation in the methylenetetrahydrofolate reductase gene: an additional risk factor for neural-tube defects? Am J Hum Genet. 1998;62(5):1044-51. 10.1086/301825 PMC1377082 9545395

[16] Coppedè F, Marini G, Bargagna S, Stuppia L, Minichilli F, Fontana I, et al. Folate gene polymorphisms and the risk of Down syndrome pregnancies in young Italian women. Am J Med Genet A. 2006;140(10):1083-91. 10.1002/ajmg.a.31217 16596679

[17] Coppedè F, Colognato R, Bonelli A, Astrea G, Bargagna S, Siciliano G, et al. Polymorphisms in folate and homocysteine metabolizing genes and chromosome damage in mothers of Down syndrome children. Am J Med Genet A. 2007;143A(17):2006-15. 10.1002/ajmg.a.31886 17702010

[18] Scazzone C, Acuto S, Guglielmini E, Campisi G, Bono A. Methionine synthase reductase (MTRR) A66G polymorphism is not related to plasma homocysteine concentration and the risk for vascular disease. Exp Mol Pathol. 2009;86(2):131-3. 10.1016/j.yexmp.2009.01.014 19348062

[19] Kandi V, Vadakedath S. Effect of DNA Methylation in Various Diseases and the Probable Protective Role of Nutrition: A Mini-Review. Cureus. 2015;7(8):e309. 10.7759/cureus.309 PMC4582005 26430583

[20] Llorca J, Prieto-Salceda D, Combarros O, Dierssen-Sotos T, Berciano J. [Competing risks of death and Hardy-Weinberg equilibrium in case-control studies of gene-disease association]. Gac Sanit. 2005;19(4):321-4. 10.1157/13078032 16050969

[21] Xu J, Turner A, Little J, Bleecker ER, Meyers DA. Positive results in association studies are associated with departure from Hardy-Weinberg equilibrium: hint for genotyping error? Hum Genet. 2002;111(6):573-4. 10.1007/s00439-002-0819-y 12516594

[22] Wittke-Thompson JK, Pluzhnikov A, Cox NJ. Rational inferences about departures from Hardy-Weinberg equilibrium. Am J Hum Genet. 2005;76(6):967-86. 10.1086/430507 PMC1196455 15834813

[23] Wu JB, Zhai JF, Yang J. Role of methylenetetrahydrofolate reductase C677T and A1298C polymorphisms in polycystic ovary syndrome risk. Genet Mol Res. 2016;15(4). 10.4238/gmr15048570 28081274

[24] Szafarowska M, Segiet A, Jerzak MM. Methylenotetrahydrololate reductase A1298C and C677T polymorphisms and adverse pregnancy outcome in women with PCOS. Neuro Endocrinol Lett. 2016;37(2):141-6. 27179578

[25] Carlus SJ, Sarkar S, Bansal SK, Singh V, Singh K, Jha RK, et al. Is MTHFR 677 C>T Polymorphism Clinically Important in Polycystic Ovarian Syndrome (PCOS)? A Case-Control Study, Meta-Analysis and Trial Sequential Analysis. PLoS One. 2016;11(3):e0151510. 10.1371/journal.pone.0151510 PMC4794143 26983014

[26] Qi Q, Zhang H, Yu M, Wang X, Wang Z, Xu L, et al. [Association of methylenetetrahydrofolate reductase gene polymorphisms with polycystic ovary syndrome]. Zhonghua Yi Xue Yi Chuan Xue Za Zhi. 2015;32(3):400-4. 10.3760/cma.j.issn.1003-9406.2015.03.022 26037361

[27] Idali F, Zareii S, Mohammad-Zadeh A, Reihany-Sabet F, Akbarzadeh-Pasha Z, Khorram-Khorshid HR, et al. Plasminogen activator inhibitor 1 and methylenetetrahydrofolate reductase gene mutations in iranian women with polycystic ovary syndrome. Am J Reprod Immunol. 2012;68(5):400-7. 10.1111/aji.12002 22882325

[28] Karadeniz M, Erdogan M, Zengi A, Eroglu Z, Tamsel S, Olukman M, et al. Methylenetetrahydrofolate reductase C677T gene polymorphism in Turkish patients with polycystic ovary syndrome. Endocrine. 2010;38(1):127-33. 10.1007/s12020-010-9370-0 20960113

[29] Choi SW, Gu BH, Ramakrishna S, Park JM, Baek KH. Association between a single nucleotide polymorphism in MTHFR gene and polycystic ovary syndrome. Eur J Obstet Gynecol Reprod Biol. 2009;145(1):85-8. 10.1016/j.ejogrb.2009.04.013 19427093

[30] Palep-Singh M, Picton HM, Yates ZR, Barth J, Balen AH. Polycystic ovary syndrome and the single nucleotide polymorphisms of methylenetetrahydrofolate reductase: a pilot observational study. Hum Fertil (Camb). 2007;10(1):33-41. 10.1080/14647270600950157 17454207

[31] Sukla KK, Raman R. Association of MTHFR and RFC1 gene polymorphism with hyperhomocysteinemia and its modulation by vitamin B12 and folic acid in an Indian population. Eur J Clin Nutr. 2012;66(1):111-8. 10.1038/ejcn.2011.152 21878957

[32] Vink JM, Sadrzadeh S, Lambalk CB, Boomsma DI. Heritability of polycystic ovary syndrome in a Dutch twin-family study. J Clin Endocrinol Metab. 2006;91(6):2100-4. 10.1210/jc.2005-1494 16219714

[33] Ewens KG, Stewart DR, Ankener W, Urbanek M, McAllister JM, Chen C, et al. Family-based analysis of candidate genes for polycystic ovary syndrome. J Clin Endocrinol Metab. 2010;95(5):2306-15. 10.1210/jc.2009-2703 PMC2869537 20200332

[34] Barbieri RL. Polycystic ovary syndrome. ACP Medicine. 2010;1-15.

[35] Cupisti S, Häberle L, Dittrich R, Oppelt PG, Reissmann C, Kronawitter D, et al A. Smoking is associated with increased free testosterone and fasting insulin levels in women with polycystic ovary syndrome, resulting in aggravated insulin resistance. Fertil Steril. 2010;94(2):673-7. 10.1016/j.fertnstert.2009.03.062 19394003

